# Supplementary material for: Evaluation of the Audicor Acoustic Cardiography Device as a Diagnostic Tool in Horses with Mitral or Aortic Valve Insufficiency
Source: Animals (Basel). 2024 Jan 21;14(2):331. doi: 10.3390/ani14020331 (PMC10812805; doi:10.3390/ani14020331)
Supplement: Supplementary file 1 [file animals-14-00331-s001.zip › Suppl_Figure S2_Linear Regression and Bland Altman analysis.pdf]

Figure S2: Linear regression and Bland-Altman analysis

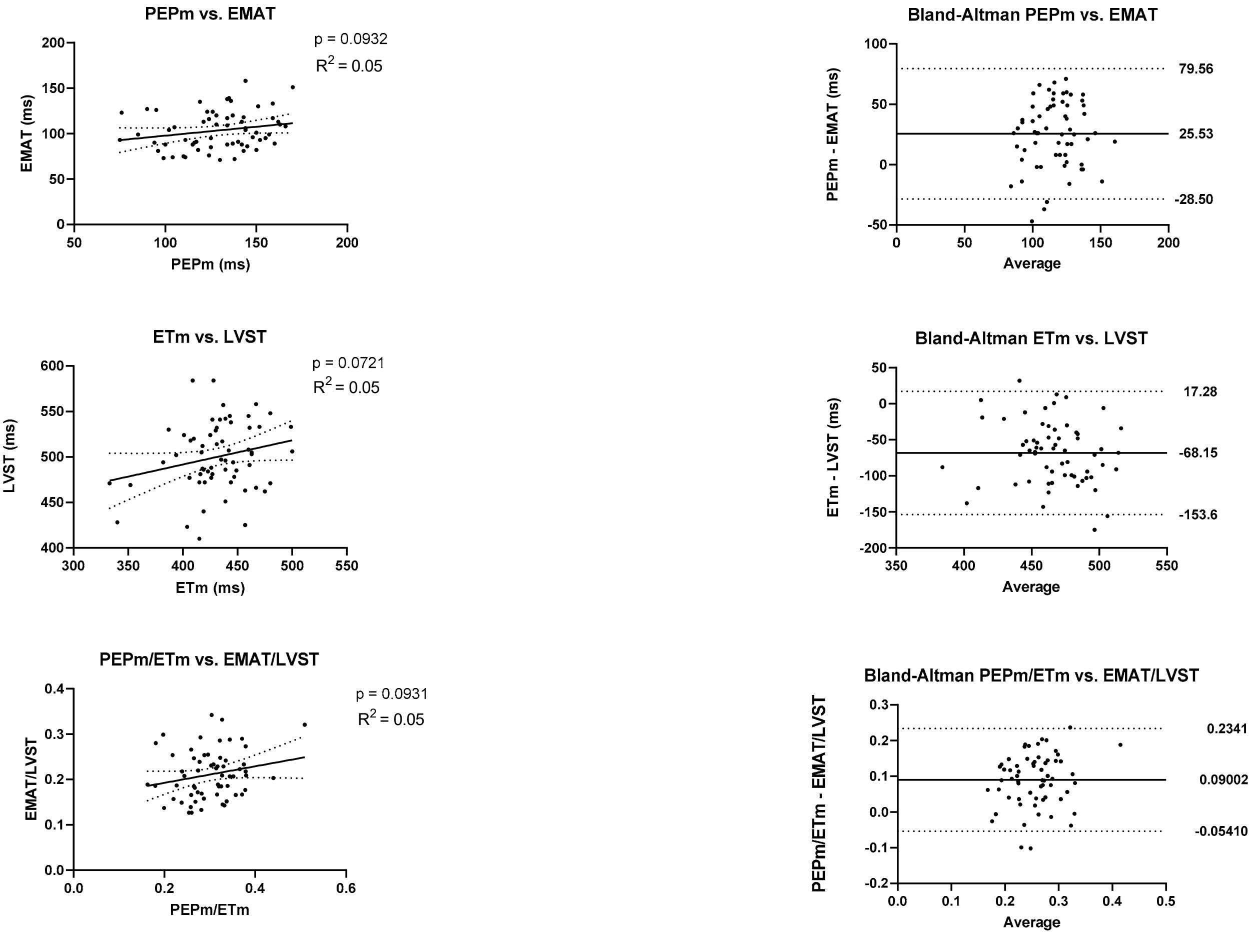

Left, linear regression analyses interrogating the relationship between the Audicor® snapshot variables (i.e., EMAT, LVST, EMAT/LVST) and the corresponding echocardiographic variables (i.e., PEP_m, ET_m, PEP_m/ET_m). The solid line represents the regression line, the dotted lines represent the 95% confidence intervals of the regression line. The P values and the coefficients of determination (R^2) are displayed for each regression analysis.

Right, Bland-Altman analyses interrogating the mean bias and 95% limits of agreement for Audicor® snapshot variables (i.e., EMAT, LVST, EMAT/LVST) and the corresponding echocardiographic variables (i.e., PEP_m, ET_m, PEP_m/ET_m). The solid lines represent the mean bias, the dotted lines illustrate the lower and the upper 95% limit of agreement.
