# Supplementary material for: Evaluation of the Audicor Acoustic Cardiography Device as a Diagnostic Tool in Horses with Mitral or Aortic Valve Insufficiency
Source: Animals (Basel). 2024 Jan 21;14(2):331. doi: 10.3390/ani14020331 (PMC10812805; doi:10.3390/ani14020331)
Supplement: Supplementary file 1 [file animals-14-00331-s001.zip › Suppl_Scoring sheet S1_AR Severity Score_.pdf]

Scoring sheet S1: AR Severity Score

1. **Left ventricular size:** Left ventricular volume at end diastole, measured in a right-parasternal long-axis view using single-plane Simpson's method of discs, normalized to a body weight of 500 kg using methods of allometric scaling (also see Supplementary Material Table S1).

**LVIV_d (500) [1]**	**Points**
≤ 1300 mL	1
1301 – 1800 mL	2
1801 – 2300 mL	3
> 2300 mL	4

2. **Jet size:** Size of the regurgitant jet (as visualized by Color Flow Doppler) relative to the left ventricular outflow tract (LVOT) in a right-parasternal long-axis view.

**Jet size [2,3]**	**Points**
≤ ¼ **LVOT** Area	1
≤ ½ **LVOT** Area	2
≤ ¾ **LVOT** Area	3
> ¾ **LVOT** Area	4

3. **Subjective evaluation of the left ventricle:** Size and shape of the left ventricle in a right-parasternal long-axis view.

**Criteria evaluated [2]**	**Points**
• Normal ventricle, no enlargement<br>• Normal apex shape	1
• Normal ventricle, mild enlargement<br>• Normal apex shape	2
• Large ventricle, moderate enlargement<br>• Round apex shape	3
• Large ventricle, moderate enlargement<br>• Globoid apex shape	4
• Large ventricle, severe enlargement<br>• Very globoid apex shape	5

4. **Total score**

**Point Score**	**Severity**
3 – 5	Mild
6 – 9	Moderate
10 – 13	Severe

References:

1. Berthoud, D.; Schwarzwald, C.C. Echocardiographic assessment of left ventricular size and systolic function in Warmblood horses using linear measurements, area-based indices, and volume estimates: A retrospective database analysis. *J. Vet. Intern. Med.* **2021**, *35*, 504-520.
2. Ven, S.; Decloedt, A.; Van Der Vekens, N.; De Clercq, D.; van Loon, G. Assessing aortic regurgitation severity from 2D, M-mode and pulsed wave Doppler echocardiographic measurements in horses. *The Veterinary Journal* **2016**, *210*, 34-38.
3. Young, L.; Rogers, K.; Wood, J. Heart murmurs and valvular regurgitation in thoroughbred racehorses: epidemiology and associations with athletic performance. *J. Vet. Intern. Med.* **2008**, *22*, 418-426.
