# Supplementary material for: Evaluation of the Audicor Acoustic Cardiography Device as a Diagnostic Tool in Horses with Mitral or Aortic Valve Insufficiency
Source: Animals (Basel). 2024 Jan 21;14(2):331. doi: 10.3390/ani14020331 (PMC10812805; doi:10.3390/ani14020331)
Supplement: Supplementary file 1 [file animals-14-00331-s001.zip › Suppl_Scoring sheet S2_MR Severity Score.pdf]

Scoring sheet S2: MR Severity Score

1. **Left atrial size:** Maximum left atrial area at end systole, measured in a right-parasternal long-axis view optimized to image the left atrium, normalized to a body weight of 500 kg using methods of allometric scaling (also see Supplementary Material Table S1).

LAA_{max} (500) [1]	Points
≤ 100 cm^2	1
101 – 120 cm^2	2
121 – 140 cm^2	3
> 140 cm^2	4

2. **Jet size:** Size of the regurgitant jet (as visualized by Color Flow Doppler) relative to the left atrial area in a right-parasternal long-axis four chamber view, optimized to image the left atrium.

Jet size compared to LAA [2]	Points
≤ ¼ **LA** Area	1
≤ ½ **LA** Area	2
≤ ¾ **LA** Area	3
> ¾ **LA** Area	4

3. **Subjective evaluation:** Size and shape of the left atria in a right-parasternal long-axis view, optimized to image the left atrium.

Criteria evaluated	Points
• Normal LA, no enlargement<br>• Normal shape	1
• Mild enlargement<br>• Normal shape	2
• Moderate enlargement<br>• Rounded shape	3
• Severely enlarged atrium<br>• Very round	4

4. Total score

Point Score	Severity
3 – 5	Mild
6 – 9	Moderate
10 – 12	Severe

References:

1. Huesler, I.M.; Mitchell, K.J.; Schwarzwald, C.C. Echocardiographic Assessment of Left Atrial Size and Function in Warmblood Horses: Reference Intervals, Allometric Scaling, and Agreement of Different Echocardiographic Variables. *J. Vet. Intern. Med.* **2016**, *30*, 1241-1252.
2. Young, L.; Rogers, K.; Wood, J. Heart murmurs and valvular regurgitation in thoroughbred racehorses: epidemiology and associations with athletic performance. *J. Vet. Intern. Med.* **2008**, *22*, 418-426.
