# Supplementary material for: Evaluation of the Audicor Acoustic Cardiography Device as a Diagnostic Tool in Horses with Mitral or Aortic Valve Insufficiency
Source: Animals (Basel). 2024 Jan 21;14(2):331. doi: 10.3390/ani14020331 (PMC10812805; doi:10.3390/ani14020331)
Supplement: Supplementary file 1 [file animals-14-00331-s001.zip › Suppl_Table S1_List of echocardiographic variables.pdf]

Table S1: List of echocardiographic variables.

**Measurements of left atrial (LA) size and function (2DE, right-parasternal long-axis view of left atrium and left ventricle, optimized to image the LA**		
LAD_{max}	cm	Internal left atrial diameter measured at the widest distance parallel to the mitral valve annulus at the time of maximum atrial filling (one frame before mitral valve opening)
LAD_{max} (500)	cm	LAD_{max} corrected to a BWT of 500kg
LAA_{max}	cm^2	Internal left atrial area measured during maximum atrial filling (one frame before mitral valve opening)
LAA_{max} (500)	cm^2	LAA_{max} corrected to a BWT of 500kg
LAA_a	cm^2	Internal left atrial area measured at the onset of active atrial contraction (at the onset of the electrocardiographic P wave)
LAA_{min}	cm^2	Internal left atrial area measured during minimum atrial filling (at the time of closure of the mitral valve)
Active LA FAC	%	Active LA fractional area change (Active LA FAC = LAA_a – LAA_{min}/LAA_a)
**Measurements of left atrial size (2DE, right-parasternal short-axis view at the aortic valve level, optimized for the left atrium and left-atrial appendage)**		
$LA_{sx}A_{max}$ (500)	cm^2	LA area at end-systole (closure of aortic valve) corrected to a BWT of 500kg
**Linear measurements of LV size and function (M-mode, right-parasternal short-axis view of the left ventricle at the chordal level)**		
$LVID_d$, $LVID_s$	cm	Left ventricular internal diameter at end-diastole (at the onset of the electrocardiographic QRS complex) and at peak-systole (at the time at which the left ventricular internal lumen is narrowest)
$LVID_d$ (500)	cm	$LVID_d$ corrected to a BWT of 500kg
LV FS	%	Left ventricular fractional shortening (LV FS = ($LVID_d$ – $LVID_s$) / $LVID_d$ × 100)
LAD_{max}/$LVID_d$	-	LAD_{max}-to-$LVID_d$ ratio
**Volumetric estimates of LV size and function (2DE, right-parasternal long-axis view of left atrium and left ventricle, optimized to image the LV, single-plane Simpson's model of discs)**		
LVIV_d, LVIV_s	mL	Left ventricular internal volume at end-diastole and at peak systole
LVIV_d (500)	mL	LVIV_d corrected to a BWT of 500kg
LV EF	%	Left ventricular ejection fraction (LV EF = (LVIV_d – LVIV_s) / LVIV_d × 100)
**Variables characterizing left atrial and left ventricular mechanical function (pulsed-wave tissue Doppler imaging of the left ventricular free wall, right-parasternal short-axis view of the left ventricle at the chordal level)**		
PEP_m	msec	Pre-ejection period
ET_m	msec	Ejection time
PEP_m/ET_m	-	PEP_m:ET_m ratio
$IVCT_m$	msec	Isovolumetric contraction time (onset S1 to onset S_m)
$IVRT_m$	msec	Isovolumetric relaxation time (onset E1 to onset E_m)
IMP_m	-	Index of myocardial performance (IMP_m = ($IVCT_m$ + $IVRT_m$) / ET_m)
E_m	cm/sec	Maximum wall motion velocity during early diastole (corresponding to passive ventricular filling)
A_m	cm/sec	Maximum wall motion velocity during late diastole (corresponding to atrial contraction)
E_m/A_m	-	E_m:A_m ratio
