# Supplementary material for: Evaluation of the Audicor Acoustic Cardiography Device as a Diagnostic Tool in Horses with Mitral or Aortic Valve Insufficiency
Source: Animals (Basel). 2024 Jan 21;14(2):331. doi: 10.3390/ani14020331 (PMC10812805; doi:10.3390/ani14020331)
Supplement: Supplementary file 1 [file animals-14-00331-s001.zip › Suppl_Table S2_List of Audicor acoustic cardiography variables.pdf]

Table S2: List of Audicor® acoustic cardiography variables.

Variable	Unit	Definition
HR	min^{-1}	Heart rate
QRS	msec	QRS duration
QTc	msec	Rate-corrected QT interval (Bazett's equation)<br>QTc = QT/√RR, where QT = QT interval and RR = preceding RR interval
EMAT	msec	Electromechanical activation time = Time interval between onset of the electrocardiographic Q wave and the first heart sound (S1)
LVST	msec	Left ventricular systolic time = Time interval between the first (S1) and the second heart sound (S2)
EMATc	%	Rate-corrected EMAT = EMAT expressed as % of the length of the corresponding cardiac cycle (electrocardiographic RR interval)
LVSTc	%	Rate-corrected LVST = LVST expressed as % of the length of the corresponding cardiac cycle (electrocardiographic RR interval)
S3	-	Power (= function of the intensity and persistence) of the third heart (S3) sound, expressed as an index ranging between 0 and 10
S4	-	Power (= function of the intensity and persistence) of the fourth heart (S4) sound, expressed as an index ranging between 0 and 10
SDI	-	Systolic Dysfunction Index = Function of QRS duration, QT interval, EMATc and intensity of S3, expressed as an index ranging between 0 and 10.<br>SDI = f([QRS] × [QT] × [S3] × [EMATc])
