# Supplementary material for: Evaluation of the Audicor Acoustic Cardiography Device as a Diagnostic Tool in Horses with Mitral or Aortic Valve Insufficiency
Source: Animals (Basel). 2024 Jan 21;14(2):331. doi: 10.3390/ani14020331 (PMC10812805; doi:10.3390/ani14020331)
Supplement: Supplementary file 1 [file animals-14-00331-s001.zip › Suppl_Table S3_Description of the study sample.pdf]

Table S3: Description of the study sample.

						Dominant insufficiency			Additional insufficiencies			
**Group**	**No**	**Breed**	**Age (y)**	**Sex**	**Body-weight (kg)**	**Valve**	**Murmur Grade (1-6/6)**	**Severity score**	**Valve**	**Murmur Grade (1-6/6)***	**Severity**	**Audible Heart sounds**
**HC**	1	Warmblood	18	mc	505	-	-	-	Aorta<br>Mitral<br>Pulmonic	-<br>-<br>-	trivial<br>trivial<br>trivial	S1, S2
**HC**	2	Warmblood	6	mc	500	-	-	-	Mitral	-	trace	S1, S2
**HC**	3	Standardbred	14	mc	470	-	-	-	Pulmonic<br>Tricuspid	-<br>-	trace<br>trace	S1, S2, S3
**HC**	4	Standardbred	14	mc	540	-	-	-	Mitral<br>Pulmonic<br>Tricuspid	-<br>-<br>-	trivial<br>trivial<br>trivial	S1, S2, S3
**HC**	5	Warmblood	13	mc	520	-	-	-	Aortic<br>Tricuspid	-<br>-	trivial<br>trace	S1, S2
**HC**	6	Warmblood	18	mc	540	-	-	-				S1, S2
**HC**	7	Warmblood	5	f	530	-	-	-				S1, S2, S3
**HC**	8	Warmblood	6	mc	530	-	-	-				S1, S2
**HC**	9	Warmblood	15	mc	580	-	-	-	Tricuspid	3/6	mild	S1, S2
**HC**	10	Warmblood	18	f	575	-	-	-	Aortic	-	trivial	S1, S2
**HC**	11	Warmblood	15	f	500	-	-	-				S1, S2
**HC**	12	Warmblood	6	f	525	-	-	-				S1, S2
**HC**	13	Warmblood	15	f	575	-	-	-				S1, S2
**HC**	14	Warmblood	7	f	575	-	-	-				S1, S2
**HC**	15	Warmblood	9	f	500	-	-	-				S1, S2
**HC**	16	Warmblood	10	f	575	-	-	-				S1, S2
**HC**	17	Warmblood	12	mc	550	-	-	-				
**MI**	1	Warmblood	10	mc	570	Mitral	3/6	mild	-		-	S1, S2
**MI**	2	Warmblood	18	f	370	Mitral	3/6	mild	-		-	S1, S2

**MI**	3	Warmblood	20	mc	470	Mitral	4/6	mild	-		-	S1, S2
**MI**	4	Warmblood	20	f	540	Mitral	4/6	mild	-		-	S1, S2
**MI**	5	Warmblood	28	m	530	Mitral	4/6	mild	Aorta	-	trivial	S1, S2, S4
**MI**	6	Warmblood	12	f	620	Mitral	4/6	mild	Pulmonic	-	trivial	S1, S2
**MI**	7	Warmblood	9	f	600	Mitral	3/6	mild	Aorta<br>Pulmonic	-<br>-	trivial<br>trivial	S1, S2
**MI**	8	Warmblood	19	f	570	Mitral	3/6	mild	Aorta<br>Pulmonic	-<br>-	trivial<br>trivial	S1, S2, S4
**MI**	9	Warmblood	17	mc	600	Mitral	3/6	mild	Aortic<br>Pulmonic	-<br>-	trivial<br>trivial	S, S2, S4
**MI**	10	Warmblood	19	f	650	Mitral	3/6	mild	Aortic<br>Pulmonic	-<br>-	trivial<br>trivial	S1, S2
**MI**	11	Andalusian	14	m	510	Mitral	4/6	mild	Aortic<br>Tricuspid	-<br>2/6	trivial<br>trace	S1, S2
**MI**	12	Warmblood	8	mc	610	Mitral	3/6	mild	Aorta<br>Pulmonic	-<br>-	trivial<br>trivial	S1, S2
**MI**	13	Warmblood	5	f	480	Mitral	3/6	mild	Aorta<br>Pulmonic	-<br>2/6	trivial<br>mild	S1, S2, S4
**MI**	14	Warmblood	18	f	690	Mitral	2/6	mild	Aortic<br>Tricuspid	-<br>-	trace<br>trivial	S1, S2, S4
**MI**	15	Warmblood	9	f	520	Mitral	2/6	mild	Aortic<br>Pulmonic<br>Tricuspid	-<br>-<br>2/6	trivial<br>trivial<br>mild	S1, S2
**MI**	16	Warmblood	9	mc	605	Mitral	3/6	mild	Aortic<br>Pulmonic<br>Tricuspid	-<br>-<br>2/6	trivial<br>trace<br>mild	S1, S2
**MI**	17	Warmblood	7	f	530	Mitral	3/6	mild	Aortic<br>Pulmonic<br>Tricuspid	-<br>-<br>-	trace<br>trace<br>trivial	S1, S2, S4
**MI**	18	Warmblood	16	mc	540	Mitral	3/6	mild	Aortic<br>Pulmonic<br>Tricuspid	-<br>-<br>-	trace<br>trace<br>trivial	S1, S2
**MI**	19	Arabian	24	mc	440	Mitral	3/6	mild	Aortic<br>Pulmonic<br>Tricuspid	-<br>-<br>-	trivial<br>trivial<br>trivial	S1, S2
**MI**	20	Warmblood	16	mc	580	Mitral	4/6	moderate	Pulmonic	-	trivial	S1, S2

**MI**	21	Warmblood	5	f	500	Mitral	6/6	moderate	Aortic	2/6	mild	S1, S2
**MI**	22	Warmblood	7	mc	560	Mitral	4/6	moderate	Aortic<br>Pulmonic	-<br>-	trivial<br>trivial	S1, S2
**MI**	23	Warmblood	7	mc	580	Mitral	4/6	moderate	Aortic<br>Tricuspid	-<br>-	mild<br>trivial	S1, S2
**MI**	24	Warmblood	18	mc	630	Mitral	6/6	moderate	Aortic<br>Pulmonic<br>Tricuspid	2/6	trace<br>trivial<br>trivial	S1, S2
**MI**	25	Warmblood	14	mc	565	Mitral	3/6	moderate	Aortic<br>Pulmonic<br>Tricuspid	-<br>-<br>-	trivial<br>trivial<br>trivial	S1, S2
**MI**	26	Warmblood	8	f	500	Mitral	4/6	moderate	Aortic<br>Pulmonic<br>Tricuspid	-<br>-<br>-	mild<br>trivial<br>mild	S1, S2
**MI**	27	Warmblood	4	m	600	Mitral	4/6	moderate	Aortic<br>Pulmonic<br>Tricuspid	-<br>-<br>2/6	trivial<br>mild<br>trivial	S1, S2
**MI**	28	Warmblood	20	f	540	Mitral	4/6	moderate	Aortic<br>Pulmonic<br>Tricuspid		mild<br>trivial<br>trivial	S1, S2, S4
**AI**	1	Freiberger	16	mc	580	Aorta	3/6	mild	-		-	S1, S2
**AI**	2	Warmblood	10	f	520	Aorta	3/6	mild	Pulmonic	-	mild	S1, S2
**AI**	3	Warmblood	20	f	590	Aorta	4/6	mild	Mitral	-	trivial	S1, S2
**AI**	4	Warmblood	11	mc	640	Aorta	4/6	moderate	-		-	S1, S2
**AI**	5	Friesian	15	mc	540	Aorta	3/6	moderate	-		-	S1, S2
**AI**	6	Warmblood	19	f	585	Aorta	4/6	moderate	-		-	S1, S2
**AI**	7	Warmblood	16	f	640	Aorta	5/6	moderate	Mitral	-	trivial	S1, S2, S4
**AI**	8	Warmblood	19	mc	540	Aorta	4/6	moderate	Mitral	-	trace	S1, S2
**AI**	9	Warmblood	22	mc	605	Aorta	4/6	moderate	Pulmonic	-	trivial	S1, S2
**AI**	10	Warmblood	21	mc	705	Aorta	4/6	moderate	Mitral	3/6	mild	S1, S2
**AI**	11	Warmblood	15	mc	510	Aorta	2/6	moderate	Mitral<br>Tricuspid		trivial<br>trivial	S1, S2
**AI**	12	Arabian	25	f	520	Aorta	4/6	moderate	Mitral<br>Pulmonic	2/6	mild<br>trivial	S1, S2

									Tricuspid		mild	
**AI**	13	Warmblood	27	f	530	Aorta	6/6	severe	-		-	S1, S2
**AI**	14	Warmblood	18	mc	575	Aorta	4/6	severe	-		-	S1, S2, S4
**AI**	15	Arabian	22	mc	400	Aorta	3/6	severe	-		-	S1, S2
**AI**	16	Warmblood	22	mc	590	Aorta	4/6	severe	Mitral	-	mild	S1, S2
**AI**	17	Friesian	27	mc	520	Aorta	6/6	severe	Mitral<br>Pulmonic	-<br>-	trace<br>trivial	S1, S2
**AI**	18	Arabian	21	m	390	Aorta	4/6	severe	Mitral<br>Pulmonic<br>Tricuspid	2/6<br>-<br>-	moderate<br>mild<br>trivial	S1, S2, S4

f=female; m=male; mc=male castrated

*Murmur grade = -, no murmur audible on auscultation and reported findings are based on color flow Doppler
