# Supplementary figures and images for: Evaluation of the Audicor Acoustic Cardiography Device as a Diagnostic Tool in Horses with Mitral or Aortic Valve Insufficiency
Source: Animals (Basel). 2024 Jan 21;14(2):331. doi: 10.3390/ani14020331 (PMC10812805; doi:10.3390/ani14020331)

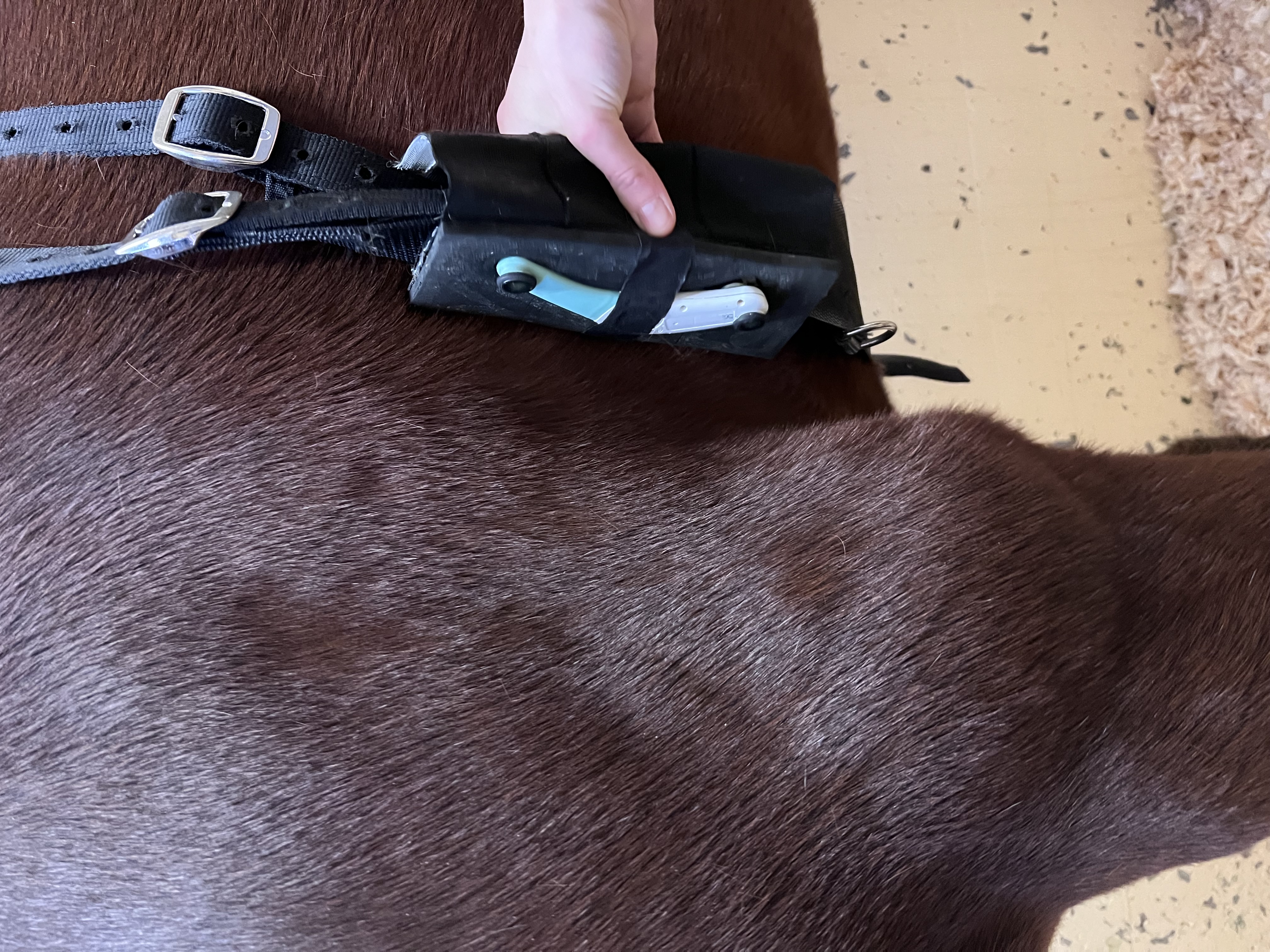

Supplement: Supplementary file 1 [file animals-14-00331-s001.zip › Suppl_Figure S1_Audicor device attached.JPG]
